# Supplementary material for: CTCF regulates wild-type and recombinant AAV gene expression by shaping viral chromatin
Source: bioRxiv. 2026 Jun 3:2026.06.03.729793. Preprint. [Version 1] doi: 10.64898/2026.06.03.729793 (PMC13252373; doi:10.64898/2026.06.03.729793)
Supplement: 1 [file NIHPP2026.06.03.729793v1-supplement-1.pdf]

bioRxiv preprint doi: https://doi.org/10.64898/2026.06.03.729793; this version posted June 3, 2026. The copyright holder for this preprint (which was not certified by peer review) is the author/funder, who has granted bioRxiv a license to display the preprint in perpetuity. It is made available under a CC-BY 4.0 International license.

Supplemental Figure S1: wtAAV2 *in silico* CBEs.

Results of JASPAR informatics resource *in silico* prediction analysis of CBEs on wtAAV2. Table shows the position and strand. Consensus score is calculated by Position-Specific Weight Matrix (PWM) (score ≥ 0.8 threshold for a strong candidate binding site).

Supplemental Figure S2: Comparison of rAAV2 and rAAV2^{CBE} transduction efficiencies.

A) Fold change of percentage of GFP positive single cells from flow cytometry analysis of rAAV2^{CBE} relative to rAAV2 at indicated MOI. Fold change shown in red. Dots represent individual calculated values with bar showing mean and error bars represent standard error mean (SEM).

Supplemental Figure S3: AAV2-CBE in rAAV8 and rAAV9.

A) Comparison of rAAV2 and rAAV2/8 transductions in HEK293T cells at indicated MOI. rAAV2 (teal), rAAV2^{CTCF} (green), rAAV2/8 (pink), rAAV2/8^{CTCF} (orange). B) Quantification of flowcytometry percentage of single cells that are GFP positive over increasing MOI comparing rAAV2/9 (dark purple) and rAAV2/9^{CTCF} (light purple). G) Median Fluorescent Intensity of GFP in arbitrary units from flowcytometry over increasing MOI. Red dashed line represents average mock

bioRxiv preprint doi: https://doi.org/10.64898/2026.06.03.729793; this version posted June 3, 2026. The copyright holder for this preprint (which was not certified by peer review) is the author/funder, who has granted bioRxiv a license to display the preprint in perpetuity. It is made available under a CC-BY 4.0 International license.

MFI. HEK293T cells 24 hpt (n=3). Error bars represent standard error mean (SEM). Statistic p-value ns (not shown) >0.5, * <0.03, ** <0.002, *** <0.0002, **** <0.0001.

bioRxiv preprint doi: https://doi.org/10.64898/2026.06.03.729793; this version posted June 3, 2026. The copyright holder for this preprint (which was not certified by peer review) is the author/funder, who has granted bioRxiv a license to display the preprint in perpetuity. It is made available under a CC-BY 4.0 International license.

Figure S1

Genome location	Consensus score	Start	Strand	Predicted sequence
p5	0.850	231	-	ACCACATGGTGTCGC
	0.846	229	-	TGACCACATGGTGTCGCAA
p19	0.814	877	+	AACGGTTGGTGGCGC
VP	0.801	3029	-	ACCCCCAAGGGGTGC

bioRxiv preprint doi: https://doi.org/10.64898/2026.06.03.729793; this version posted June 3, 2026. The copyright holder for this preprint (which was not certified by peer review) is the author/funder, who has granted bioRxiv a license to display the preprint in perpetuity. It is made available under a CC-BY 4.0 International license.

Figure S2

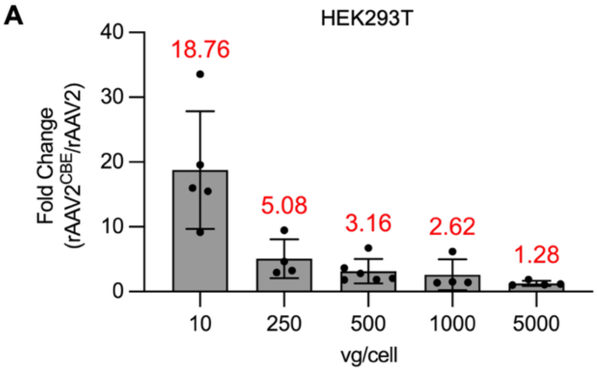

1267

bioRxiv preprint doi: https://doi.org/10.64898/2026.06.03.729793; this version posted June 3, 2026. The copyright holder for this preprint (which was not certified by peer review) is the author/funder, who has granted bioRxiv a license to display the preprint in perpetuity. It is made available under a CC-BY 4.0 International license.

Figure S3

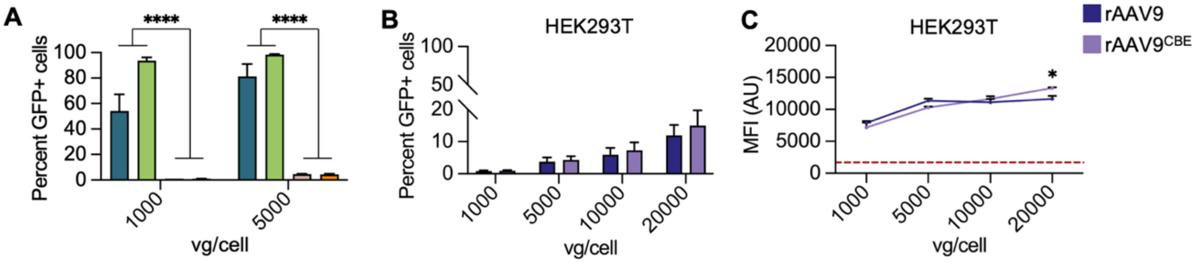

1268
